# Conservative oxygen therapy in critically ill and perioperative period of patients with sepsis-associated encephalopathy

**DOI:** 10.3389/fimmu.2022.1035298

**Published:** 2022-10-19

**Authors:** Yun Li, Lina Zhao, Yang Yu, Kai Zhang, Yi Jiang, Zhiwei Wang, Keliang Xie, Yonghao Yu

**Affiliations:** ^1^ Department of Anesthesiology, Tianjin Medical University General Hospital, Tianjin, China; ^2^ Tianjin Research Institute of Anesthesiology, Tianjin, China; ^3^ Department of Critical Care Medicine, Tianjin Medical University General Hospital, Tianjin, China

**Keywords:** sepsis, sepsis-associated encephalopathy, oxygen saturation, incidence, mortality

## Abstract

**Objectives:**

Sepsis-associated encephalopathy (SAE) patients in the intensive care unit (ICU) and perioperative period are administrated supplemental oxygen. However, the correlation between oxygenation status with SAE and the target for oxygen therapy remains unclear. This study aimed to examine the relationship between oxygen therapy and SAE patients.

**Methods:**

Patients diagnosed with sepsis 3.0 in the intensive care unit (ICU) were enrolled. The data were collected from the Medical Information Mart for Intensive Care IV (MIMIC IV) database and the eICU Collaborative Research Database (eICU-CRD) database. The generalized additive models were adopted to estimate the oxygen therapy targets in SAE patients. The results were confirmed by multivariate Logistic, propensity score analysis, inversion probability-weighting, doubly robust model, and multivariate COX analyses. Survival was analyzed by the Kaplan-Meier method.

**Results:**

A total of 10055 patients from eICU-CRD and 1685 from MIMIC IV were included. The incidence of SAE patients was 58.43%. The range of PaO_2_ (97-339) mmHg, PaO_2_/FiO_2_ (189-619), and S_P_O_2_≥93% may reduce the incidence of SAE, which were verified by multivariable Logistic regression, propensity score analysis, inversion probability-weighting, and doubly robust model estimation in MIMIC IV database and eICU database. The range of PaO_2_/FiO_2_ (189-619) and S_P_O_2_≥93% may reduce the hospital mortality of SAE were verified by multivariable COX regression.

**Conclusions:**

SAE patients in ICU, including perioperative period, require conservative oxygen therapy. We should maintain S_P_O_2_≥93%, PaO_2_ (97-339) mmHg and PaO_2_/FiO_2_ (189-619) in SAE patients.

## Introduction

Sepsis-associated encephalopathy (SAE) refers to cognitive dysfunction that can be attributed to systemic inflammatory responses in the absence of direct infection of the central nervous system (1). The incidence of SAE is over 70% of patients admitted to the intensive care unit (ICU) (2). SAE correlates with higher mortality (50.3%), longer hospital stays, and poorer long-term outcomes (3).

Patients admitted to the ICU and perioperative period are administered supplemental oxygen as low partial pressure of arterial oxygen (PaO_2_) is detrimental. However, a high PaO_2_ correlates with increased mortality, as confirmed in previous studies (4, 5). In a medical-surgical population of adult critically ill patients, arterial oxygen saturation (S_P_O_2_) supplementation titrated to 94%-98% correlates with favorable outcomes (6). The frequent oxygen exposure above the protocol goal (PaO_2_ >80 mmHg and FiO_2_ >0.5) correlates with worse clinical outcomes in patients who develop acute respiratory distress syndrome (7). PaO_2_ between (77-220) mmHg and the PaO_2_/FiO_2_ ratio in the range of 314-788 also correlates with favorable neurologic outcomes (8). Lower or higher oxygenation targets correlate with worse patient outcomes in ICU and perioperative period.

Potentially adjusted factors contributing to SAE include acute renal failure, hyperglycemia >10 mmol/l, hypercapnia >45 mmHg, and hypernatremia >145 mmol/l (3). The relationships between lower or higher oxygen therapy targets and the incidence and survival in SAE patients remain unclear. This study aimed to assess the correlation of SpO_2,_ PaO_2_, and PaO_2_/FiO_2_ with SAE in ICU and perioperative period, and elucidate the optimal oxygen therapy targets in SAE patients.

## Materials and methods

### Study settings

We collected information on patients admitted to the ICU between 2008-2019 from the MIMIC-IV 0.4 and between 2014-2015 from the eICU-CRD v2.0 (NO. 33690380) database. MIMIC IV comprises 69619 and eICU-CRD of 200859 ICU admissions. The eICU database is a multi-center dataset. These were approved by the institutional review boards of the Massachusetts Institute of Technology and Beth Israel Deaconess Medical Center (9). The requirement for individual patient consent was waived because the project does not impact clinical care and all patient confidential information was anonymized. MIMIC-IV and eICU included the demographics, laboratory measurements, microbiology cultures diagnoses, and other patient data. The MIMIC IV database (version 1.0) is publicly available on https://physionet.org/content/mimiciv/1.0/ and the eICU is publicly available on https://eICU-crd.mit.edu/about/eICU/. The raw data were extracted by employing structure query language (SQL) with Navicat and further processed using the R software.

### Patients

Sepsis was diagnosed with an acute change in the total sequential organ failure assessment (SOFA) score ≥2 and documented or suspected infection complied with the sepsis-3.0 criteria (10). The patients with infection sites or prescriptions of antibiotics and samples of bodily fluids for microbiological culture had suspected infection. In line with the existing literature, the microbiological sample must have been collected within 24 h when the antibiotic was first administered, and at the first occurrence of microbiological sampling, the antibiotic administration would be within 72 h (11). The SOFA score was defined in the first 24 h of the admission of the patient to the ICU.

SAE in this study was defined as the Glasgow Coma Scale (GCS) <15 or diagnosed delirium (3, 12, 13). Consciousness disorder with clear causes was excluded. GCS has been established as a clinically effective tool to characterize SAE and distinguish it from sepsis (3, 14). Specifically for the sedated or postoperative patients, GCS that was evaluated before sedation or surgery was extracted.

Inclusion criteria were as follows: 1) patients aged over 18 years; 2) ICU stays with more than 24 h of oxygen therapy, and 3) patients complying with the diagnostic criteria as in sepsis 3.0.

Following were the exclusion criteria (3, 12): 1) patients with brain injury (e.g., traumatic brain injury, meningitis, encephalitis intracerebral hemorrhage, cerebral embolism, ischemic stroke, epilepsy, brain tumor or intracranial infection, and any other cerebrovascular disease); 2) mental disorders and neurological disease; 3) chronic alcohol or drug abuse; 4) metabolic encephalopathy, hepatic encephalopathy, hypertensive encephalopathy, hypoglycemic coma, and other liver disease or kidney disease that affected consciousness; 5) severe electrolyte imbalances or glycemic disturbances, including hyponatremia (<120 mmol/l), hyperglycemia (>180 mg/dl), or hypoglycemia (<54 mg/dl); 6) those without GCS assessment; 7) missing values of SpO_2_, FiO_2_, PaO_2_ or no signs of administration of supplemental oxygen. Hyponatremia, hyperglycemia, and hypoglycemia can cause metabolic encephalopathy. Patients with any of the above-mentioned conditions were excluded.

### Data collection

Patient information (e.g., age, gender, length of hospital stay, and hospital mortality), laboratory indicators, co-existing illnesses, sites of infection, microbiology types, and advanced cardiac life support (e.g., mechanical ventilation and vasopressors) were extracted as the demographic data using SQL. The laboratory indicators of the patients were collected within the first 24 h of their ICU stay, including pulse oxygen saturation (S_P_O_2_), partial pressure of carbon dioxide (PaCO_2_), partial pressure of oxygen (PaO_2_), and a fraction of inspired oxygen (FiO_2_). The coexisting illnesses were determined following the recorded International Classification of Diseases (ICD)-9 and ICD-10 codes (e.g., hypertension, diabetes, pulmonary disease, and kidney disease). Disease severity scores included the SOFA score and GCS. Only the data of the first ICU admission of the corresponding patients have been included.

### Statistical analysis

The data were analyzed using the R software. Data distributions were analyzed by the Shapiro-Wilk test. Herein, all the data exhibited skewed distributions. Continuous data (age, PaCO_2_, FiO_2_, PaO_2_, S_P_O_2_, length of hospital stay, SOFA, and GCS) were expressed as median and interquartile ranges (IQR). Other categorical data were expressed in counts and proportions. Continuous variables were examined using the non-parametric Mann-Whitney U-test. Furthermore, categorical variables were compared using the Fisher exact test.

PaO_2_ and PaO_2_/FiO_2_ show a nonlinear correlation with the incidence in SAE patients. The generalized additive models (15) were adopted to estimate the association between median PaO_2_, PaO_2_/FiO_2,_ and SAE, and the rage of PaO_2_, PaO_2_/FiO_2_ target was determined for reducing the incidence and hospital mortality among SAE patients.

The range of PaO_2_, S_P_O_2_, PaO_2_/FiO_2_ targets for incidence and hospital mortality of SAE patients as determined using multivariate Logistic, COX regression models, propensity score analysis, inversion probability-weighting, and doubly robust model for further validation of the oxygen values. An independent association between blood oxygen index levels and patients’ SAE was inferred through the doubly robust estimation method. Multivariate Logistic regression and Extreme Gradient Boosting (XGBoost) were used to create propensity score models for the 7 covariables (**Figure 3**) in sepsis patients with SAE. A cohort of inverse probability of treatment weighting (IPTW) was generated from the estimated propensity scores. Afterward, we performed a Logistic Regression on the weighted cohort to adjust for remaining unbalanced variables in the propensity score model between SAE groups and non-SAE groups, resulting in a double robust analysis. To determine whether IPTW reduced the imbalance of covariate distribution, the standardized mean difference (SMD) of the original cohort was compared with the SMD of the IPTW cohort. The Kaplan-Meier curves were analyzed using Log-rank tests.

## Results

### Baseline characteristics

**Figure 1:**
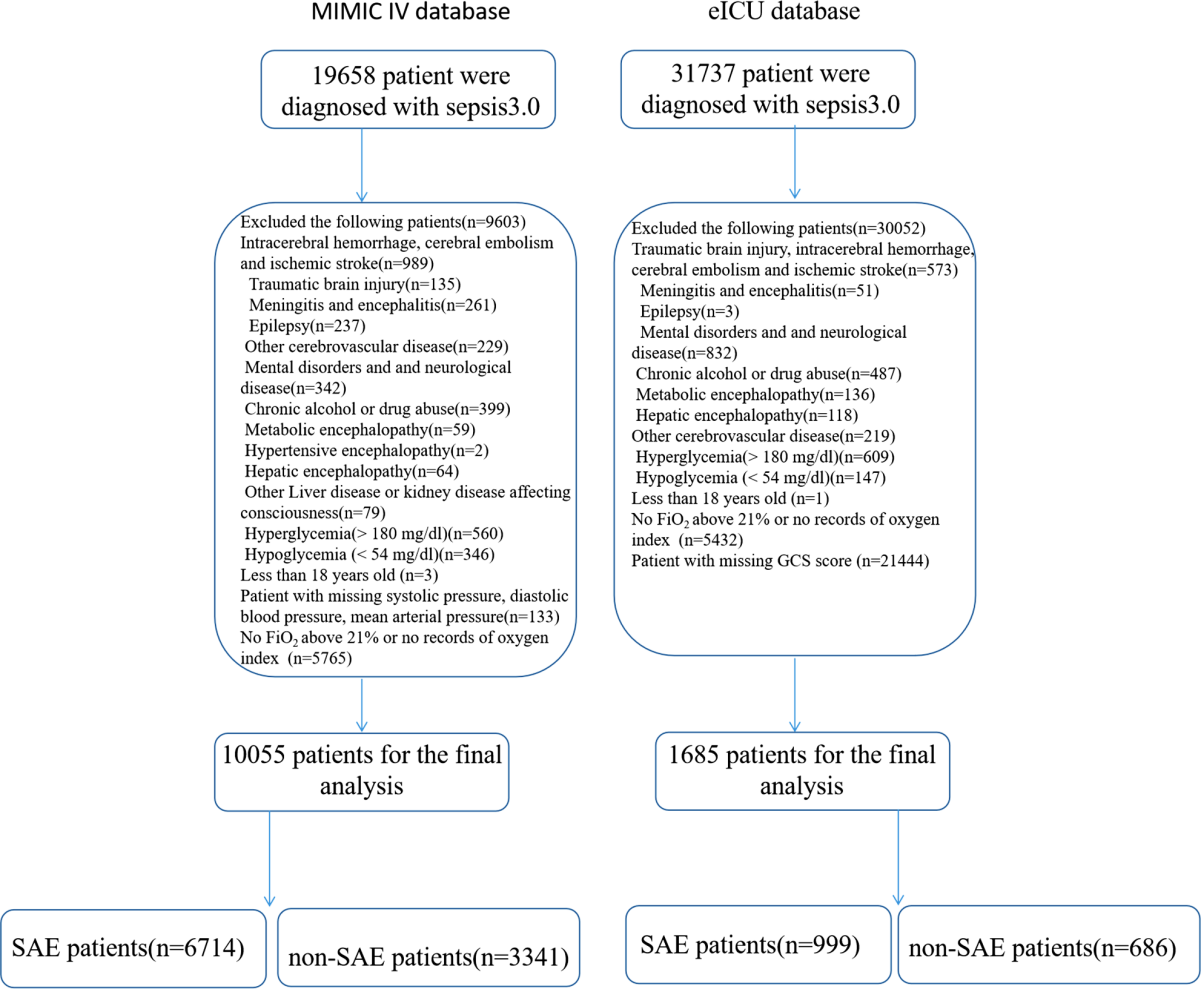
Flow chart for patient selection. ICU, intensive care unit; PaCO_2_, partial pressure of carbon dioxide; FiO_2_, the fraction of inspired oxygen.

A total of 51395 patients met the sepsis 3.0 criteria in the MIMIC IV database and the eICU database; among them, 39655 patients were excluded from the analysis due to intracerebral hemorrhage, encephalitis, brain tumor, brain injury, mental disorders, drug abuse, alcoholism, Alzheimer’s disease, metabolic encephalopathy, hepatic encephalopathy, the absence of FiO_2_ over 21%, missing records of the oxygen index, or no records of GCS scores; three patients were younger than 18 years and other conditions. A total of 11740 patients were included in the final analysis (**Figure 1**).

**Table 1** summarized the characteristics and outcomes of sepsis in the MIMIC IV database. Relative to the non-SAE group in the original cohort, patients in the SAE group were more likely to suffer from Klebsiella, Escherichia coli, or fungus infection. The SAE group showed higher respiratory rates, FiO_2_, and lower PaO_2_, S_P_O_2_, and PaO_2_/FiO_2,_ relative to the non-SAE group. Patients in the SAE group exhibited higher SOFA and hospital mortality with a longer length of hospital stay.

**Table 1:** Baseline characteristics and outcomes of sepsis patients.

	Original cohort			Match cohort		
	Non-SAE patients (n=3341)	SAE patients (n=6714)	P	Non-SAE patients (n=3327)	SAE patients (n=3327)	P
Baseline variables						
Age(years) (median [IQR])	69.00 [60.00, 77.00]	68.00[59.00, 77.00]	0.540	69.00[59.00, 77.00]	68.00[59.00, 77.00]	0.036
Gender,M (%)	2103 (62.9)	4252 (63.3)	0.722	2099 (63.1)	2105 (63.3)	0.899
Coexisting illness, (n(%))						
Hypertension	553 (16.6)	1129 (16.8)	0.760	548 (16.5)	532 (16.0)	0.618
Diabetes	558 (16.7)	1100 (16.4)	0.707	558 (16.8)	484 (14.5)	0.014
Respiration	1065 (31.9)	2015 (30.0)	0.059	1059 (31.8)	713 (21.4)	<0.001
Renal	1362 (40.8)	2754 (41.0)	0.807	1357 (40.8)	1222 (36.7)	0.001
Site of infection, (n (%))						
Urinary	200 ( 6.0)	360 ( 5.4)	0.215	195 ( 5.9)	167 ( 5.0)	0.144
Lung	185 ( 5.5)	370 ( 5.5)	0.993	182 ( 5.5)	122 ( 3.7)	0.001
Catheter	65 ( 1.9)	96 ( 1.4)	0.063	64 ( 1.9)	39 ( 1.2)	0.017
Skin and soft tissue	103 ( 3.1)	205 ( 3.1)	0.984	56 (1.7)	102 ( 3.1)	<0.001
Abdominal cavity	89 ( 2.7)	166 ( 2.5)	0.612	88 ( 2.6)	64 ( 1.9)	0.059
Microbiology type, (n (%))						
Acinetobacter baumannii	6 ( 0.2)	20 ( 0.3)	0.373	5 ( 0.2)	12 ( 0.4)	0.145
Klebsiella	161 ( 4.8)	454 ( 6.8)	<0.001	160 ( 4.8)	177 ( 5.3)	0.371
Escherichia Coli	340 (10.2)	845 (12.6)	<0.001	338 (10.2)	352 (10.6)	0.601
Pseudomonas aeruginosa	133 ( 4.0)	175 ( 2.6)	<0.001	120 ( 3.6)	117 ( 3.5)	0.895
Staphylococcus aureus	38 ( 1.1)	63 ( 0.9)	0.403	36 ( 1.1)	32 ( 1.0)	0.715
Fungus	300 ( 9.0)	1118 (16.7)	<0.001	299 ( 9.0)	302 ( 9.1)	0.932
Vital signs, (median [IQR])						
Respiratory rate (bpm)	26.00 [23.00, 31.00]	27.00[24.00, 31.00]	<0.001	26.00[23.00, 31.00]	26.00[23.00, 30.00]	0.001
S_P_O_2_, %	94.00 [92.00, 95.00]	93.00[90.00, 95.00]	<0.001	95.00[93.00, 96.00]	94.00[92.00, 95.00]	<0.001
FiO_2_, %	50.00 [40.00, 55.00]	50.00[40.00, 66.00]	<0.001	43.00[40.00, 50.00]	50.00[40.00, 55.00]	<0.001
PaO_2_, mmHg	99.00 [80.00, 129.00]	93.00[74.00, 123.00]	<0.001	118.00[95.00, 152.00]	99.00[80.00, 129.00]	<0.001
PaCO_2_, mmHg	47.00 [42.00, 52.00]	47.00[41.00, 52.00]	0.171	47.00[42.00, 52.00]	44.00[39.00, 49.00]	<0.001
PaO_2_/FiO_2_	219.00 [150.00, 264.00]	216.00 [136.70, 248.00]	<0.001	248.60 [219.00, 316.00]	219.00 [150.00, 264.00]	<0.001
Laboratory parameters (median [IQR])						
White blood cell (×10^9^ /L)	14.10 [10.70, 18.70]	14.10[10.50, 18.60]	0.58	14.10[10.70, 18.70]	13.90[10.40, 18.10]	0.078
Hemoglobin(g/dL)	9.30 [8.10, 10.70]	9.30 [8.10, 10.70]	0.278	9.30 [8.00, 10.70]	9.20 [8.00, 10.50]	0.025
Platelet (×10^9^ /L)	141.00[107.00, 201.00]	144.00[108.75, 206.00]	0.022	141.00[107.00, 201.00]	136.00[104.00, 191.00]	<0.001
Creatinine(mg/dL)	1.00 [0.80, 1.40]	1.00 [0.80, 1.40]	0.255	1.00 [0.80, 1.40]	1.00 [0.80, 1.40]	0.233
Blood urea nitrogen (mg/dL)	19.00 [14.00, 28.00]	19.00[14.00, 29.00]	0.209	19.00[14.00, 27.00]	18.00[14.00, 27.00]	0.036
Glucose(mg/dL)	133.00[113.00, 164.00]	134.00[113.00, 165.00]	0.407	133.00[112.50, 164.00]	132.00[111.00, 161.00]	0.164
Sodium (mmol/l)	140.00[137.00, 142.00]	140.00[137.00, 142.00]	0.629	140.00[137.00, 142.00]	140.00[138.00, 142.00]	0.342
Lactates (mmol/L)	1.70 [1.20, 2.40]	1.70 [1.20, 2.30]	0.003	1.70 [1.20, 2.40]	1.60 [1.20, 2.30]	0.004
The score system, (median [IQR])						
SOFA	3.00 [2.00, 5.00]	5.00 [3.00, 7.00]	<0.001	3.00 [2.00, 5.00]	5.00 [3.00, 7.00]	<0.001
GCS	15.00 [15.00, 15.00]	13.00[8.00, 14.00]	<0.001	15.00[15.00, 15.00]	13.00[8.00, 14.00]	<0.001
Mechanical ventilation, (n(%))	2575 (77.1)	5153 (76.8)	0.737	2563 (77.0)	2500 (75.1)	0.075
Use of vasopressors, (n(%))	2080 (62.3)	4036 (60.1)	0.040	2070 (62.2)	1966 (59.1)	0.010
Length of hospital stays, days (median [IQR])	2.30 [1.30, 4.90]	2.40 [1.30, 5.10]	0.142	2.30 [1.30, 4.90]	2.30 [1.30, 4.80]	0.876
Hospital mortality, (n(%))	250 ( 7.5)	914 (13.6)	<0.001	248 (7.5)	222 (6.7)	0.232

GCS, Glasgow coma scale; SOFA, sequential organ failure assessment;PaCO_2_, partial pressure of carbon dioxide; S_P_O_2_, arterial oxygen saturation; PaO_2_, partial pressure of oxygen.

### Generalized additive models to estimate the optimal oxygen therapy targets for incidence of SAE

**Figure 2:**
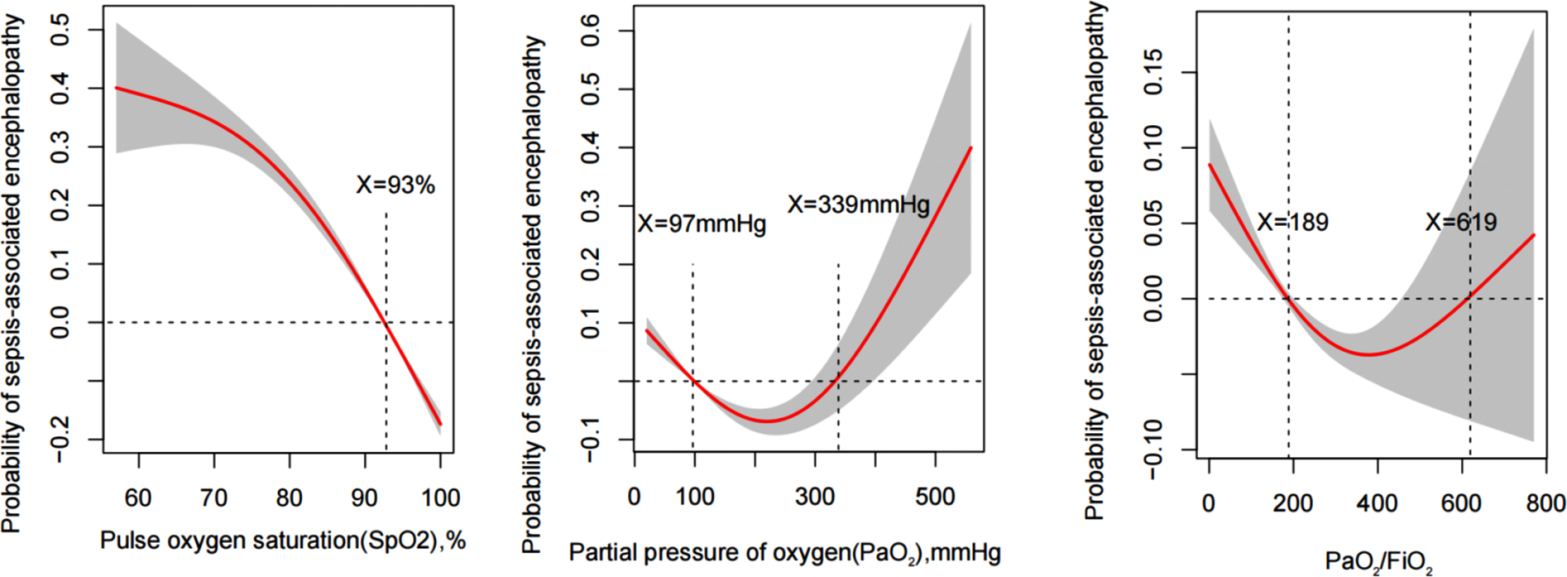
Generalized additive model plots for the median of S_P_O_2_, PaO_2_, and PaO_2_/FiO_2_ indicating the probability of sepsis-associated encephalopathy incidence.

**Figure 2** illustrated the correlation between the incidence of SAE and the median S_P_O_2_, PaO_2_, and PaO_2_/FiO_2_ determined using the generalized additive models in MIMIC IV database. The generalized additive models demonstrated a nonlinear correlation between PaO_2_ and PaO_2_/FiO_2_ with SAE incidence. S_P_O_2_<93%, PaO_2_<97 mmHg and >339 mmHg, and PaO_2_/FiO_2_<189 and >619 were associated with increased incidence of SAE, as shown in **Table 2**.

**Table 2:** Multiple models analysis of blood oxygen index to incidence in sepsis with encephalopathy.

	Models	OR	CI		P
			2.5%	97.5%	
**Internal cohort study(MIMIC database cohort study)**					
**Multivariate Logistic analysis (Original cohort)**					
S_P_O_2_≥93%		0.32	0.27	0.39	<0.001
PaO_2_/FiO_2_(189-619)		0.51	0.45	0.58	<0.001
PaO_2_(97-339)mmHg		0.57	0.47	0.68	<0.001
**Propensity score matching**					
S_P_O_2_≥93%		0.59	0.55	0.65	<0.001
PaO_2_/FiO_2_(189-619)		0.87	0.80	0.95	0.002
PaO_2_(97-339)mmHg		0.76	0.70	0.82	<0.001
**Propensity score IPW**					
S_P_O_2_≥93%		0.61	0.56	0.67	<0.001
PaO_2_/FiO_2_(189-619)		0.88	0.81	0.96	0.003
PaO_2_(97-339)mmHg		0.79	0.72	0.86	<0.001
**Doubly robust with all covariates**					
S_P_O_2_≥93%		0.85	0.82	0.87	<0.001
PaO_2_/FiO_2_(189-619)		0.96	0.93	0.98	0.002
PaO_2_(97-339)mmHg		0.92	0.90	0.95	<0.001
**External validation cohort study(eICU database cohort study)**					
**Multivariate Logistic analysis**					
S_P_O_2_≥93%		0.78	0.63	0.96	0.020
PaO_2_/FiO_2_(189-619)		0.75	0.56	0.98	0.048
PaO_2_(97-339)mmHg		0.70	0.55	0.90	0.006
**Propensity score matching**					
S_P_O_2_≥93%		0.79	0.65	0.96	0.018
PaO_2_/FiO_2_(189-619)		0.73	0.60	0.90	0.002
PaO_2_(97-339)mmHg		0.32	0.24	0.41	<0.001
**Propensity score IPW**					
S_P_O_2_≥93%		0.78	0.64	0.96	0.020
PaO_2_/FiO_2_(189-619)		0.76	0.62	0.94	0.011
PaO_2_(97-339)mmHg		0.69	0.57	0.85	0.001
**Doubly robust with all covariates**					
S_P_O_2_≥93%		0.91	0.84	0.98	0.012
PaO_2_/FiO_2_(189-619)		0.90	0.83	0.97	0.006
PaO_2_(97-339)mmHg		0.87	0.81	0.94	<0.001

PaCO_2_, partial pressure of carbon dioxide; S_P_O_2_, arterial oxygen saturation; PaO_2_, partial pressure of oxygen.

### Multivariate logistic analysis for risk factors of SAE incidence

According to the results of the generalized additive model, as shown in **Figure 2**, PaO_2_ (97-339) mmHg, PaO_2_/FiO_2_ (189-619), and S_P_O_2_≥93% may reduce the incidence of SAE. After adjusting for confounders, PaO_2_ (97-339) mmHg [odds ratio (OR): 0.566, 95% confidence interval (CI): 0.471-0.681, p<0.001], PaO_2_/FiO_2_ (189-619) [OR:0.513, 95% CI: 0.452-0.582, p<0.001], and S_P_O_2_≥93% [OR:0.324, 95% CI: 0.272-0.387, p<0.001] were identified as protective factors against SAE conduct internal assessment in the MIMIC IV database (**Supplementary Material 1**). Besides, using the eICU database for external evaluation (**Supplementary Material 2**), after adjusting for confounders, PaO_2_ (97-339) mmHg [OR: 0.703, 95% CI: 0.547-0.903, p=0.006], PaO_2_/FiO_2_ (189-619) [OR:0.750, 95% CI: 0.555-0.982, p=0.048], and S_P_O_2_≥93% [OR:0.779, 95% CI: 0.631-0.961, p=0.020] were identified as protective factors (**Supplementary Materials 1**, **2** ).

### Propensity match analysis

**Figure 3:**
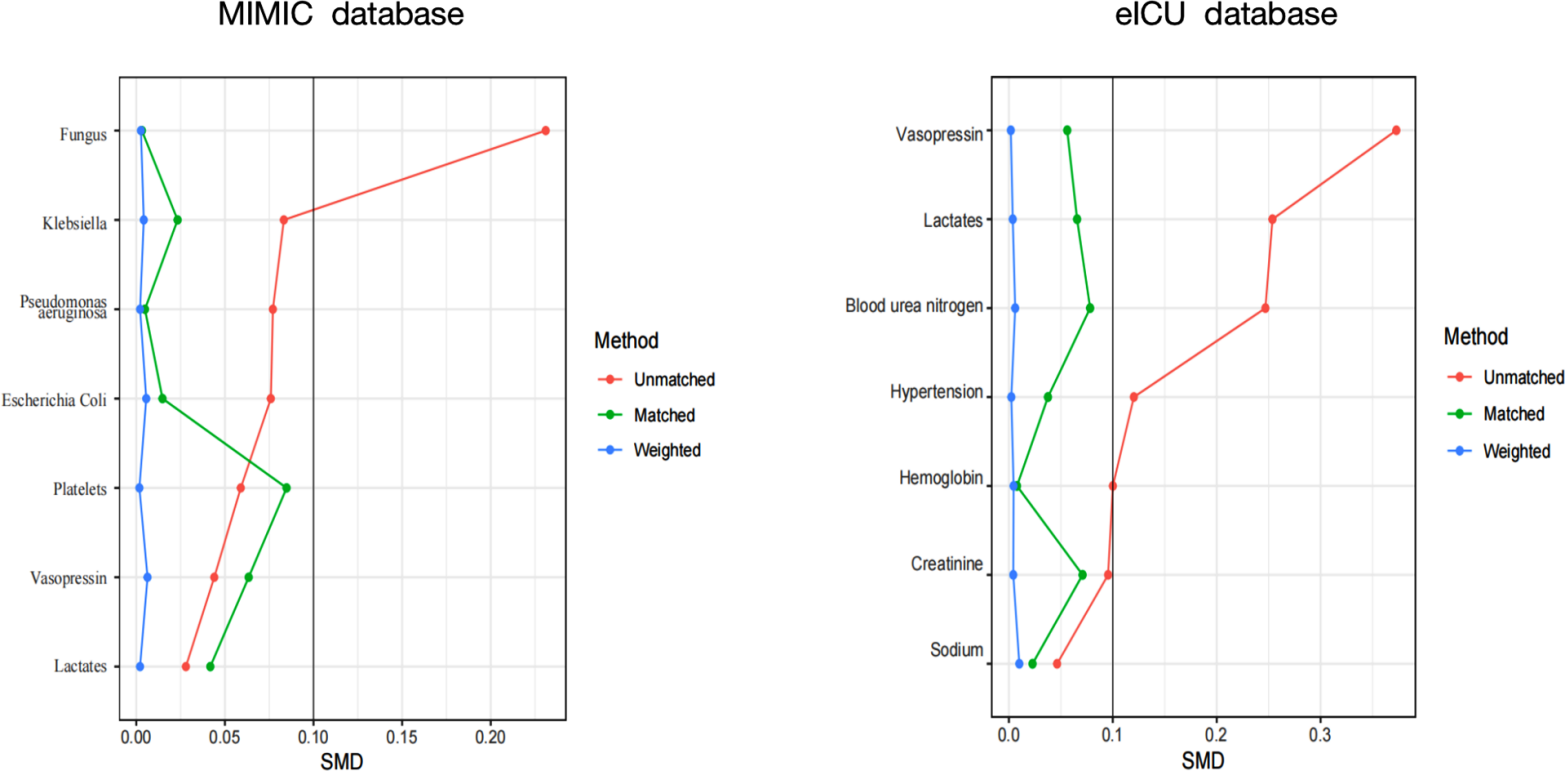
A SMD of the original cohort was compared with the SMD of the IPTW cohorts. SMD, standardized mean difference.

To further confirm the reliability of the results, the data in the MIMIC IV database is used for internal verification and the data in the eICU database is used for external verification by propensity score analysis, inversion probability-weighting, and doubly robust model. A propensity matching scoring model was constructed using 7 covariates with statistically significant differences in **Table 1** of the original cohort in the MIMIC IV database (**Figure 3**). For standardizing the differences between the SAE group and the non-SAE group, the estimated propensity scores were used. Covariates were well balanced between classes after IPTW (<0.1) (**Figure 3**). After matching the difference covariates, PaO_2_, S_P_O_2_, and PaO_2_/FiO_2_ were significantly different between the SAE group and the non-SAE group (**Table 1**). To evaluate the relationship between the PaO_2_, S_P_O_2_, and PaO_2_/FiO_2_ levels (estimated as per generalized additive model) and SAE incidence, we used propensity-matching score, proportion score IPTW and a doubly robust model with two databases to statistical analysis. The estimation models led to the same conclusion in the MIMIC IV and eICU database that S_P_O_2_≥93%, PaO_2_/FiO_2_ (189-619), and PaO_2_(97-339) mmHg were protective factors for patients with SAE (**Table 2**).

### Prognostic analyses of patients with SAE

**Figure 4:**
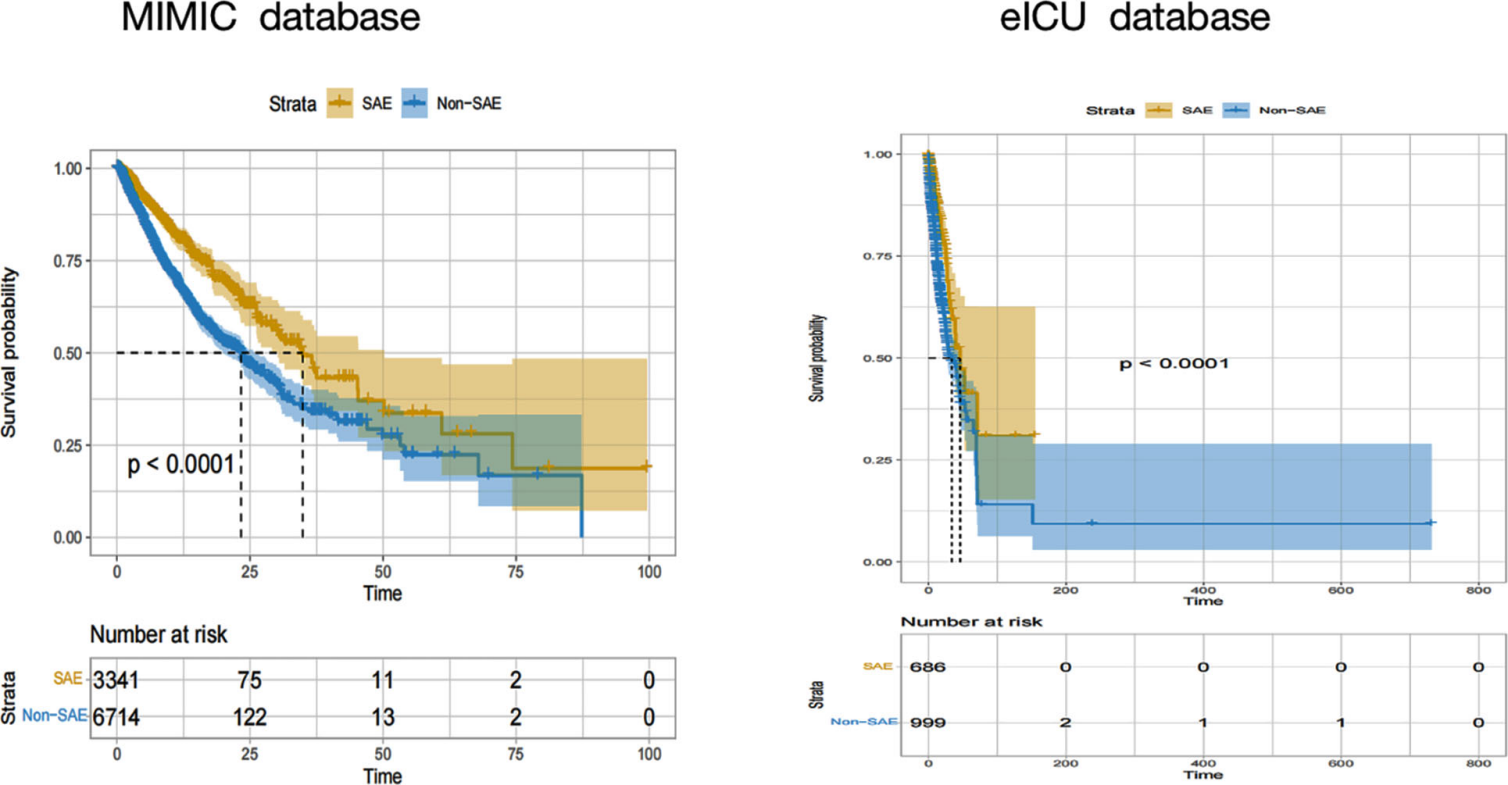
Kaplan-Meier hospital survival curves for SAE patients. SAE, sepsis-associated encephalopathy.

To further examine the effect of SAE on the prognoses of patients with sepsis, their survival was analyzed by the Kaplan-Meier method. Patients in the non-SAE group showed better survival rates than the SAE group (p<0.001) (**Figure 4**).

### Demographic and clinical characteristics of SAE

**Table 1** and **Figure 4** illustrated that SAE patients had poor prognoses. To further examine the effect of oxygen therapy on the prognoses of patients with SAE, we divided the SAE patients into the survival and non-survival groups in the MIMIC IV and eICU database. The results as listed in **Supplementary Material 3** showed that non-surviving patients had higher PaO_2_ and PaO_2_/FiO_2_.

### Multivariate COX analysis for risk factors of hospital mortality in SAE

According to the results of the generalized additive model, as shown in **Figure 2**, after adjusting for confounders, S_P_O_2_≥93% and PaO_2_/FiO_2_(189-619) were independent protective factors for SAE prognoses in the MIMIC IV and eICU database (**Table 3**; **Supplementary Materials 4**, **5**).

**Table 3:** Multiple COX regression model analysis of blood oxygen index to hospital mortality in sepsis with encephalopathy.

	Models	OR	CI		P
			2.5%	97.5%	
**Internal cohort study(MIMIC IV database cohort study)**					
S_P_O_2_≥93%		0.68	0.49	0.93	0.017
PaO_2_/FiO_2_(189-619)		0.38	0.32	0.45	<0.001
PaO_2_(97-339) mmHg		0.93	0.69	1.25	0.63
**External validation cohort study(eICU database cohort study)**					
S_P_O_2_≥93%		0.78	0.63	0.96	0.020
PaO_2_/FiO_2_(189-619)		0.75	0.56	0.98	0.048
PaO_2_(97-339) mmHg		0.70	0.55	0.90	0.006

PaCO_2_, partial pressure of carbon dioxide; S_P_O_2_, arterial oxygen saturation; PaO_2_, partial pressure of oxygen.

## Discussion

As demonstrated from the primary outcome of this cohort study, oxygen therapy may be correlated with SAE incidence. The PaO_2_ range of 97 to 339 mmHg, the PaO_2_/FiO_2_ ratio between 189 and 619, and S_P_O_2_≥93% may be correlated with reducing SAE incidence. The PaO_2_ range of 97 to 339 mmHg and the PaO_2_/FiO_2_ ratio between 189 and 619 may be correlated with reduce SAE hospital mortality. Conservative oxygen therapy should be performed for SAE patients.

As reported in existing studies, the SAE incidence can reach up to 70% (2), in line with the results of this study (58.43%), which suggested a high SAE incidence. The results of the cohort study showed that hospital mortality was 13.6% in SAE patients, the hospital mortality of SAE group was significantly higher than that of non-SAE group, and that SAE patient with a higher SOFA score and longer hospital stay. These results with consistent with the findings of a previous study (3). It has been reported that 45% of SAE patients show long-term cognitive dysfunction after hospital discharge (16). Taken together, SAE shows a high incidence and poor prognosis. However, specific treatment for SAE is rare, and early identification of potentially modifiable factors with the optimal chance of avoiding incidence, long-term cognitive dysfunction, and reducing mortality are needed. In this large cohort study, the PaO_2_ range of 97 to 339 mmHg and the PaO_2_/FiO_2_ ratio between 189 and 619 were associated with reduced incidence and hospitalization mortality of SAE. Thus, lower or higher oxygenation could induce SAE.

Hyperoxemia is correlated with neurological injury in patients with traumatic brain injury and aneurysmal subarachnoid hemorrhage (17, 18). Hyperoxemia leads to the production of reactive oxygen species, thereby destroying cells and further promoting inflammatory responses (18). Moreover, active oxygen can cause an increase in the production of free radicals of oxygen, whereby excess free radicals can stimulate the hypersensitive arterial system, resulting in vasospasm (19). Inflammatory responses, oxygen-free radicals, and vasospasm are vital mechanisms underlying SAE (20, 21). Nguyen Mai et al. propose a conceptual model of lung-brain coupling, the use of supplemental oxygen can induce cardiac arrest, neuronal injury, neuroinflammation, and memory deficits (22). The results of this study demonstrated that PaO2 >339 mmHg and PaO2/FiO2>619 may be increase the incidence of encephalopathy in patients with sepsis. Hyperoxemia has been proven to be correlated with SAE. The neurological injury in sepsis patients attributed to hyperoxia may be due to the above-mentioned reasons. A subsequent study is thus required to evaluate the underlying pathophysiological mechanism in the future (23).

Neurological injury attributed to hypoxemia has been extensively confirmed (24, 25). Kim I Chisholm et al. report that sepsis leads to increased sensitivity of cortical mitochondria to hypoxemia, and such increased sensitivity is mirrored by a decrease in cortical tissue oxygen tension in mice (26). Transcription-dependent mechanisms triggered by hypoxia and reticulum stress could activate AMP-activated protein kinase, thereby stimulating the inflammatory activities. AMP protein is vital and is stimulated by immune factors in the brain, and consequently, the MAP signaling pathway is inhibited, decreased meta apoptosis and autophagy (22, 27). Hypoxia increases the levels of lactate/pyruvate, decreases the glutathione/oxidized glutathione ratio, upregulates inflammatory cytokine cascades, activates the apoptosis pathway, all leading to the damage of the cerebral cortex and neurons (23, 28). The results of this study further demonstrated that PaO2 <97mmHg, PaO2/FiO2<189, and SPO2<93% may cause changes in consciousness among sepsis patients. To mitigate the neurological injury by hypoxia or hyperoxia and incidence of SAE, PaO2 with 97-339 mmHg, PaO2/FiO2 in the range of 189-619, and SPO2≥93% should be taken in SAE patients.

Low oxygen saturations are considered detrimental. A liberal oxygen strategy is correlated with mortality, especially in ICU patients, as oxygen is extensively used in the ICU, and patients are commonly exposed to high oxygenation (29). The correlation between exposure to hyperoxia and mortality has been reported in ICU in previous studies (30, 31). Specifically for critically ill patients or those on ventilator-assisted breathing, to reduce mortality, the assessment of optimal oxygen saturation is particularly important. Willem van den Boom et al. report that the optimal range of S_P_O_2_ is 94%–98% which is correlated with decreased hospital mortality among critically ill patients (32). The proportion of time of oxygen saturation of 95%–99% correlates with reduced mortality in critically ill patients on mechanical ventilation, as reported by Dawei Zhou et al. (33). According to Derek K Chu et al., in acutely ill adults, liberal oxygen therapy increases mortality, and oxygen saturation of 94%-96% adversely affects the patients (34). In this study, the S_P_O_2_<93% was significantly associated with hospital mortality among SAE patients. The range of PaO_2_ (97-339) mmHg and PaO_2_/FiO_2_ (189-619) are associated with lower hospital mortality in SAE patients. The findings support that SAE patients should be administrated with conservative oxygen therapy for reducing the incidence and hospital mortality.

## Limitation

First, the definition of SAE is compliance with GCS<15 score, and patients diagnosed with delirium according to ICD9 and ICD10. Though brain hemorrhage, brain trauma, and other diseases were excluded, the absence of brain computed tomography scans, magnetic resonance imaging, electroencephalogram, and other examinations to assess the nervous system, the result information bias maybe in the SAE cohort. Second, this was an observational study, and the causal correlation between oxygen therapy and the incidence and mortality of SAE could not be proved. However, the correlation between oxygen therapy and SAE was demonstrated in this large cohort study by multiple databases and Multiple statistical methods. The findings provide certain clinical reference values. Finally, due to the interrelationship between diseases, some confounding factors remained, thereby covering up or exaggerating the relationship between study factors and SAE.

## Conclusions

In conclusion, high or low PaO_2_, PaO_2_/FiO_2_ and S_P_O_2_ were correlated with the incidence of SAE. The range of PaO_2_ (97-339) mmHg, PaO_2_/FiO_2_ (189-619) and S_P_O_2_ ≥93% were identified in SAE patients in ICU and perioperative period. A reference target was provided which is expected to aid clinicians in preventing the occurrence and reducing the incidence and hospital mortality of SAE. PaO_2_ (97-339) mmHg, PaO_2_/FiO_2_ (189-619) and S_P_O_2_ ≥93% as reference targets for subsequent experiments.

## Data availability statement

Publicly available datasets were analyzed in this study. This data can be found here: The MIMIC IV database (version 1.0) is publicly available at https://mimic-iv.mit.edu/and the eICU database is publicly available at https://eicu-crd.mit.edu/about/eicu/. Any researcher who adheres to the data use requirements is permitted access to these databases. The codes are available at https://github.com/MIT-LCP/mimic-iv.

## Author contributions

YL, LZ, KX, and YHY conceived the central ideas of the study. YY, KZ, YJ, and ZW collected the data. YL and LZ wrote the first draft of the manuscript. KX and YHY revised the paper, worked on the English, and drafted the final version of the manuscript. All authors contributed to the article and approved the submitted version.

## Funding

This work was supported by the grant from the Medical and Health Science and Technology Plan Project of Inner Mongolia Autonomous Region Health Commission (202202326); Natural Science Foundation of Inner Mongolia Autonomous Region (2022MS08002); Science and Technology Support Key Program Affiliated to the Key Research and Development Plan of Tianjin Science and Technology Project (18YFZCSY00560); Natural Science Foundation of China (81772043, 81971879, 82072150); Tianjin Key Medical Discipline (Specialty) Construction Project (TJYXZDXK-036A).

## Conflict of interest

The authors declare that the research was conducted in the absence of any commercial or financial relationships that could be construed as a potential conflict of interest.

## Publisher’s note

All claims expressed in this article are solely those of the authors and do not necessarily represent those of their affiliated organizations, or those of the publisher, the editors and the reviewers. Any product that may be evaluated in this article, or claim that may be made by its manufacturer, is not guaranteed or endorsed by the publisher.

## Abbreviations

SAE, sepsis-associated encephalopathy; PaCO_2_, partial pressure of carbon dioxide; FiO_2_, fraction of inspired oxygen; PaO_2_, partial pressure of oxygen; SpO_2_ pulse oxygen saturation

arterial oxygen saturation; SOFA, sequential organ failure assessment; GCS, Glasgow coma scale; ICU, intensive care unit; MIMIC-IV, Medical Information Mart for Intensive Care IV.
