# Supplementary material for: Conservative oxygen therapy in critically ill and perioperative period of patients with sepsis-associated encephalopathy
Source: Front Immunol. 2022 Oct 19;13:1035298. doi: 10.3389/fimmu.2022.1035298 (PMC9626799; doi:10.3389/fimmu.2022.1035298)
Supplement: Supplementary file 3 [file DataSheet_3.docx]

rhc <- read.csv ("/Users/mac/Desktop/eicu.CSV", header = T)#)

# 待统计的协变量：

vars <- c("age","gender","gaoxueya","tangniaobing","lung","Uri0ry..infection","lung.infection","Catheter.related","Skin.and.soft.tissue","Abdomi0l.cavity",

"baoman", "feike","dachang","tonglv","jinpu","Fungus","pco2_max",

"resp_rate_max","spo2_min","po2_min","FiO2","pao2fio2",

"wbc_max","hemoglobin_min","platelets_min","creatinine_max","bun_max",

"glucose_max.x","sodium_max","Lac","vasopressin",

"sofa","gcs_min","ventdurations","los","hospital_expire_flag","re0l")

catVar<-c("gender","gaoxueya","tangniaobing","lung","Uri0ry..infection","lung.infection","Catheter.related","Skin.and.soft.tissue","Abdomi0l.cavity","baoman",

"feike","dachang","tonglv","jinpu","Fungus","vasopressin",

"ventdurations","re0l","hospital_expire_flag")

#分类变量

#分类变量)

## Construct a table

tabUnmatched <- CreateTableOne(vars = vars, strata = "GROUP", data = rhc, test = FALSE,factorVars = catVar)

## Show table with SMD

print(tabUnmatched, smd = TRUE)

## Fit model

psModel <- glm(formula =GROUP~gaoxueya+re0l+Uri0ry..infection+Abdomi0l.cavity+wbc_max+

hemoglobin_min+creatinine_max+bun_max+sodium_max+Lac+

vasopressin,

family = binomial(link ="logit"),

data = rhc)

## Predicted probability of being assigned to RHC

rhc$pRhc <- predict(psModel, type = "response")

## Predicted probability of being assigned to no RHC

rhc$pNoRhc <- 1 - rhc$pRhc

## Predicted probability of being assigned to the treatment actually assigned (either RHC or no RHC)

rhc$pAssign <- NA

rhc$pAssign[rhc$GROUP== 1] <- rhc$pRhc[rhc$GROUP== 1]

rhc$pAssign[rhc$GROUP== 0] <- rhc$pNoRhc[rhc$GROUP== 0]

## Smaller of pRhc vs pNoRhc for matching weight

rhc$pMin <- pmin(rhc$pRhc, rhc$pNoRhc)

listMatch <- Match(Tr = (rhc$GROUP== 1), # Need to be in 0,1

## logit of PS,i.e., log(PS/(1-PS)) as matching scale

X = log(rhc$pRhc / rhc$pNoRhc),

## 1:1 matching

M = 2,

## caliper = 0.2 * SD(logit(PS))

caliper = 0.2,

replace = FALSE,

ties = TRUE,

version = "fast")

# determining if balance exists in any unmatched dataset and in matched datasets

mb <- MatchBalance(psModel$formula, data=rhc, match.out=listMatch, nboots=50)

rhcMatched <- rhc[unlist(listMatch[c("index.treated","index.control")]), ]

## Construct a table

tabMatched <- CreateTableOne(vars = vars, strata = "GROUP", data = rhcMatched, test = T,factorVars = catVar)

## Show table with SMD

print(tabMatched, smd = TRUE)

nonnormalvars = c('age','wbc_max','hemoglobin_min','spo2_min','po2_min','FiO2','pao2fio2',

'platelets_min','creatinine_max','bun_max','glucose_max.x','sodium_max',

'Lac','sofa','gcs_min','resp_rate_max','pco2_max',

'los')

tab1<-print(tabMatched, nonnormal = nonnormalvars)

write.csv(tab1,file = "/Users/mac/Desktop/tab1.csv")

：

##the inverse probability of treatment weighting，IPTW）

##the standardized mortality ratio weighting，SMRW）,

## Matching weight

rhc$mw <- rhc$pMin / rhc$pAssign

# IPTW:

rhc$mw1=ifelse(rhc$GROUP==1,1/(rhc$pRhc),1/(1-rhc$pRhc))

## Weighted data

rhcSvy <- svydesign(ids = ~ 1, data = rhc, weights = ~ mw)

## Construct a table (This is a bit slow.)

tabWeighted <- svyCreateTableOne(vars = vars, strata = "GROUP", data = rhcSvy, test = FALSE)

## Show table with SMD

print(tabWeighted, smd = TRUE)

library(data.table)

## Construct a data frame containing variable name and SMD from all methods

dataPlot <- data.table(variable = rownames(ExtractSmd(tabUnmatched)),

Unmatched = ExtractSmd(tabUnmatched),

Matched = ExtractSmd(tabMatched),

Weighted = ExtractSmd(tabWeighted))

colnames(dataPlot) <- c("variable","Unmatched","Matched","Weighted")

## Create long-format data for ggplot2

dataPlotMelt <- melt(data = dataPlot,

id.vars = c("variable"),

variable.name = "Method",

value.name = "SMD")

## Order variable names by magnitude of SMD

varNames <- as.character(dataPlot$variable)[order(dataPlot$Unmatched)]

## Order factor levels in the same order

dataPlotMelt$variable <- factor(dataPlotMelt$variable,

levels = varNames)

## Plot using ggplot2

ggplot(data = dataPlotMelt, mapping = aes(x = variable, y = SMD,

group = Method, color = Method)) +

geom_line() +

geom_point() +

geom_hline(yintercept = 0.1, color = "black", size = 0.1) +

coord_flip() +

theme_bw() + theme(legend.key = element_blank())

####library(data.table)

## Construct a data frame containing variable name and SMD from all methods

dataPlot <- data.table(variable = rownames(ExtractSmd(tabUnmatched)),

Unmatched = ExtractSmd(tabUnmatched),

Matched = ExtractSmd(tabMatched),

Weighted = ExtractSmd(tabWeighted))

colnames(dataPlot) <- c("variable","Unmatched","Matched","Weighted")

## Create long-format data for ggplot2

dataPlotMelt <- melt(data = dataPlot,

id.vars = c("variable"),

variable.name = "Method",

value.name = "SMD")

## Order variable names by magnitude of SMD

varNames <- as.character(dataPlot$variable)[order(dataPlot$Unmatched)]

## Order factor levels in the same order

dataPlotMelt$variable <- factor(dataPlotMelt$variable,

levels = varNames)

##（hazard ratio,HR）[95% CI)]和pvalue。

## Unmatched model (unadjsuted)

glmUnmatched <- glm(formula = (GROUP== "1") ~氧合指数,

family = binomial(link = "logit"),

data = rhc)

## Matched model

glmMatched <- glm(formula = (GROUP== "0") ~氧合指数,

family = binomial(link = "logit"),

data = rhcMatched)

## Weighted model

glmWeighted <- svyglm(formula = (GROUP== "1") ~氧合指数,

family = binomial(link = "logit"),

design = rhcSvy)

## Show results together

resTogether <- list(Unmatched = ShowRegTable(glmUnmatched, printToggle = FALSE),

Matched = ShowRegTable(glmMatched, printToggle = FALSE),

Weighted = ShowRegTable(glmWeighted, printToggle = FALSE))

print(resTogether, quote = FALSE)

install.packages("drgee")

library(drgee)

library(car)

library(carData)

SLID<-read.csv ("/Users/mac/Desktop/eicu.CSV", header = T)#

fit <- drgee(exposure = "SBP",

oformula = AKI~ age+gender+tangniaobing+lung+xinzang+

Uri0ry..infection+lung.infection +Catheter.related+Skin.and.soft.tissue+Abdomi0l.cavity +baoman+feike+

dachang+tonglv+jinpu+Fungus+heart_rate_max+resp_rate_max+

wbc_max+hemoglobin_min+glucose_max.x+sodium_max+potassium_max+Lac+ sofa +

gcs_min+ventdurations+Atrialfibrillation,

iaformula = ~ age,

olink = "log",

estimation.method = "o",

data = SLID)

##

fit <- drgee(oformula = GROUP~gaoxueya+re0l+Uri0ry..infection+Abdomi0l.cavity+wbc_max+

hemoglobin_min+creatinine_max+bun_max+sodium_max+Lac+

vasopressin,

eformula =氧合指数~gaoxueya+re0l+Uri0ry..infection+Abdomi0l.cavity+wbc_max+

hemoglobin_min+creatinine_max+bun_max+sodium_max+Lac+

vasopressin,

olink = "log",

elink = "logit",

estimation.method = "dr",

data = SLID)

resTogether <- list(Unmatched = ShowRegTable(fit, printToggle = FALSE))

print(resTogether, quote = FALSE)

install.packaes('mgcv')

library(nlme)

library(mgcv)

dat<-read.csv("/Users/mac/Desktop/mimic.CSV", header = T)#)

mod<-gam(GROUP~s(po2_min,k=3),data=dat)

summary(mod)

plot(mod,pages=1,scheme=1,unconditional=TRUE,ylab="Probability of Sepsis-associated disorders of consciousness",col="red",lty=1,lwd=2)

lines(x=1:1000,y=rep(0,1000),lty=2)

lines(x=rep(96.7,800),y=seq(-0.3,0.2,length.out=800),lty=2)

lines(x=rep(338.7,600),y=seq(-0.2,0.2,length.out=600),lty=2)

text(x=100,y=0.02,"X=97")

text(x=300,y=0.02,"X=339")

locator(1)

library(data.table)

mydata=as.data.frame(fread("mydata.csv"))

library("survival")

library("survminer")

fit <- survfit(Surv(Survival.months,OS) ~ as.factor(Surgery), data = mydata) #KM分析

summary(fit)

ggsurvplot(fit,pval = T)
