# Supplementary material for: Conservative oxygen therapy in critically ill and perioperative period of patients with sepsis-associated encephalopathy
Source: Front Immunol. 2022 Oct 19;13:1035298. doi: 10.3389/fimmu.2022.1035298 (PMC9626799; doi:10.3389/fimmu.2022.1035298)
Supplement: Supplementary file 4 [file Table_1.docx]

| **Supplementary materials1** Multivariate Logistic analysis of risk factors to incidence of patients with SAE in the MIMIC database | | | | | |
| --- | --- | --- | --- | --- | --- |
|  |  | P | OR | 95.0% CI | |
|  |  |  |  | Lower | Upper |
| Microbiology type, (n (%)) |  |  |  |  |  |
|  | Klebslella | 0.641 | 1.050 | 0.855 | 1.290 |
|  | Escherichia Coli | 0.650 | 1.035 | 0.893 | 1.199 |
|  | Pseudomonas aeruginosa | 0.001 | 0.661 | 0.515 | 0.848 |
|  | Fungus | <0.001 | 1.551 | 1.337 | 1.671 |
| Respiratory rate (bpm) |  | 0.001 | 1.012 | 1.005 | 1.799 |
| PaO_2_ (97-339)mmHg |  | <0.001 | 0.566 | 0.471 | 0.681 |
| S_P_O_2_≥93% |  | <0.001 | 0.324 | 0.272 | 0.387 |
| FiO_2_, % |  | <0.001 | 1.021 | 1.018 | 1.023 |
| PaO_2_/FiO_2_(189-619) |  | <0.001 | 0.513 | 0.452 | 0.582 |
| Platelet (×109 /L) |  | 0.112 | 1.000 | 1.000 | 1.001 |
| Lactates (mmol/L) |  | <0.001 | 0.895 | 0.867 | 0.923 |
| Use of vasopressors |  | <0.001 | 0.732 | 0.666 | 0.805 |
| SOFA |  | <0.001 | 1.344 | 1.315 | 1.373 |
| S_P_O_2_: arterial oxygen saturation; PaO_2_: partial pressure of oxygen; FiO_2_: the fraction of inspired oxygen; SOFA: sequential organ failure assessmen. | | | | | |
