# Supplementary material for: Conservative oxygen therapy in critically ill and perioperative period of patients with sepsis-associated encephalopathy
Source: Front Immunol. 2022 Oct 19;13:1035298. doi: 10.3389/fimmu.2022.1035298 (PMC9626799; doi:10.3389/fimmu.2022.1035298)
Supplement: Supplementary file 5 [file Table_2.docx]

**Supplementary materials 2** Multivariate logistic analysis of risk factors to incidence

of patients with SAE in the eICU database

|  | P | OR | 95.0% CI | |
| --- | --- | --- | --- | --- |
|  |  |  | Lower | Upper |
| Renal disease | 0.295 | 1.150 | 0.885 | 1.493 |
| Urinary infection | 0.021 | 1.510 | 1.064 | 2.143 |
| Abdominal cavity infection | 0.243 | 1.646 | 0.713 | 3.802 |
| S_P_O_2 ≥_93% | 0.020 | 0.779 | 0.631 | 0.961 |
| FiO_2_, % | <0.001 | 3.397 | 1.982 | 5.824 |
| PaO_2_ (97-339)mmHg | 0.006 | 0.703 | 0.547 | 0.903 |
| PaO_2_/FiO_2_ (189-619) | 0.048 | 0.750 | 0.555 | 0.982 |
| PaCO_2_ | 0.297 | 0.996 | 0.988 | 1.004 |
| White blood cell | 0.315 | 0.996 | 0.987 | 1.004 |
| Hemoglobin | 0.106 | 0.968 | 0.931 | 1.007 |
| Creatinine | 0.004 | 0.895 | 0.831 | 0.964 |
| Blood urea nitrogen | 0.017 | 1.007 | 1.001 | 1.013 |
| Sodium | 0.835 | 0.999 | 0.994 | 1.005 |
| Lactates (mmol/L) | <0.001 | 0.895 | 0.867 | 0.923 |
| SOFA | <0.001 | 1.173 | 1.123 | 1.224 |
| Use of vasopressors | <0.001 | 0.732 | 0.666 | 0.805 |

SOFA: sequential organ failure assessment;PaCO_2_: partial pressure of carbon dioxide;

S_P_O_2_: arterial oxygen saturation; PaO_2_: partial pressure of oxygen.
