# Supplementary material for: Conservative oxygen therapy in critically ill and perioperative period of patients with sepsis-associated encephalopathy
Source: Front Immunol. 2022 Oct 19;13:1035298. doi: 10.3389/fimmu.2022.1035298 (PMC9626799; doi:10.3389/fimmu.2022.1035298)
Supplement: Supplementary file 6 [file Table_3.docx]

**Supplementary material 3** Baseline characteristics and outcomes of SAE patients in the MIMIC database and eICU database

|  | MIMICIV database | |  | eICU database | | |
| --- | --- | --- | --- | --- | --- | --- |
|  | Survival  patients  (n=5800) | Non-Survival patients  (n=914) | P | Survival  patients  (n=680) | Non-Survival patients  (n=319) | P |
| Baseline variables |  |  |  |  |  |  |
| Age(years) (median [IQR]) | 68.00 [59.00, 77.00] | 70.00[60.25, 80.00] | <0.001 | 65.50[54.00, 78.00] | 72.00[61.50, 81.00] | <0.001 |
| Gender,M (%) | 3711 (64.0) | 541 ( 59.2) | 0.006 | 330 (48.5) | 145 (45.5) | 0.401 |
| Coexisting illness, (n(%)) | | | |  |  |  |
| Hypertension | 1014 (17.5) | 115 ( 12.6) | <0.001 | 134 ( 19.7) | 65 ( 20.4) | 0.871 |
| Diabetes | 848 (14.6) | 252 ( 27.6) | <0.001 | 93 ( 13.7) | 54 ( 16.9) | 0.209 |
| Respiration | 1733 (29.9) | 282 ( 30.9) | 0.577 | 51 (7.5) | 28 (8.8) | 0.567 |
| Renal | 2321 (40.0) | 433 ( 47.4) | <0.001 | 246 ( 36.2) | 162 (50.8) | <0.001 |
| Site of infection, (n (%)) | | |  |  |  |  |
| Urinary | 311 ( 5.4) | 49 (5.4) | 1.000 | 85 ( 12.5) | 45 ( 14.1) | 0.547 |
| Lung | 285 ( 4.9) | 85 (9.3) | <0.001 | 63 (9.3) | 32 (10.0) | 0.788 |
| Catheter | 74 ( 1.3) | 22 ( 2.4) | 0.011 | 0 ( 0) | 0( 0) | 1.000 |
| Skin and soft tissue | 179 ( 3.1) | 26 ( 2.8) | 0.771 | 44 (6.5) | 16 (5.0) | 0.448 |
| Abdominal cavity | 136 ( 2.3) | 30 (3.3) | 0.114 | 16 (2.4) | 12 (3.8) | 0.293 |
| Microbiology type, (n (%)) | | | |  |  |  |
| Acinetobacter baumannii | 16 ( 0.3) | 4 (0.4) | 0.612 | 35 (5.1) | 19 (6.0) | 0.706 |
| Klebsiella | 381 ( 6.6) | 73 ( 8.0) | 0.13 | 14 (2.1) | 6 (1.9) | 1.000 |
| Escherichia Coli | 741 (12.8) | 104 ( 11.4) | 0.258 | 43 (6.3) | 15 (4.7) | 0.381 |
| Pseudomonas aeruginosa | 153 ( 2.6) | 22 ( 2.4) | 0.768 | 21 (3.1) | 8 (2.5) | 0.759 |
| Staphylococcus aureus | 59 ( 1.0) | 4 (0.4) | 0.132 | 58 (8.5) | 25 (7.8) | 0.805 |
| Fungus | 956 (16.5) | 162 ( 17.7) | 0.374 | 9 (1.3) | 4 (1.3) | 1.000 |
| Vital signs, (median [IQR]) | | | |  |  |  |
| Respiratory rate (bpm) | 27.00 [23.00, 31.00] | 28.00[25.00, 33.00] | <0.001 | 21.00[18.00, 24.00] | 21.00[19.00, 27.00] | <0.001 |
| S_P_O_2_, % | 93.00 [91.00, 95.00] | 90.00[82.00, 93.00] | <0.001 | 93.00[90.00, 93.00] | 93.00[90.00, 93.00] | 0.335 |
| FiO_2_, % | 50.00 [40.00, 60.00] | 69.00[49.00, 100.00] | <0.001 | 44 [36, 60] | 60 [40, 100] | <0.001 |
| PaO_2_, mmHg | 95.00 [76.00, 126.00] | 77.00[62.00, 101.00] | <0.001 | 109.00[78.40, 115.00] | 93.80[72.15, 110.00] | 0.002 |
| PaCO_2_, mmHg | 46.00 [40.00, 52.00] | 47.00[41.00, 54.75] | <0.001 | 44.0[36.00, 45.73] | 43.3[33.40, 48.00] | 0.635 |
| PaO_2_/FiO_2_ | 219.00[150.00, 264.00] | 216.00[136.70, 248.00] | <0.001 | 222.50[149.60, 311.40] | 165.00[109.00, 254.25] | <0.001 |
| Laboratory parameters (median [IQR]) | | |  |  |  |  |
| White blood cell (×10^9^ /L) | 14.00 [10.50, 18.50] | 14.20[10.33, 19.40] | 0.533 | 14.10 [9.60, 19.70] | 15.80[10.25, 21.20] | 0.062 |
| Hemoglobin(g/dL) | 9.40 [8.20, 10.70] | 9.20 [7.90, 10.80] | 0.088 | 10.05 [8.60, 11.90] | 9.40 [8.10, 11.05] | <0.001 |
| Platelet (×10^9^ /L) | 143.00[109.00, 203.00] | 152.50[103.00, 227.00] | 0.108 | 187.50[124.00, 241.00] | 167.00[94.00, 242.50] | 0.014 |
| Creatinine(mg/dL) | 1.00 [0.80, 1.40] | 1.20 [0.80, 1.90] | <0.001 | 1.30 [0.90, 2.20] | 1.70 [1.03, 2.65] | 0.001 |
| Blood urea nitrogen (mg/dL) | 19.00 [14.00, 27.00] | 24.50[16.00, 41.00] | <0.001 | 30.00[19.00, 45.00] | 36.00[24.00, 56.50] | <0.001 |
| Glucose(mg/dL) | 132.00[112.00, 161.00] | 145.00[117.00, 187.00] | <0.001 | 115.00[94.00, 144.25] | 111.00[85.00, 141.00] | 0.039 |
| Sodium (mmol/l) | 140.00[137.00, 142.00] | 140.00[137.00, 142.75] | 0.069 | 140.00[137.00, 144.00] | 140.00[137.00, 144.00] | 0.703 |
| Lactates (mmol/L) | 1.60 [1.20, 2.30] | 1.70 [1.20, 2.30] | 0.106 | 3.40 [1.70, 3.40] | 3.40 [2.30, 5.40] | <0.001 |
| The score system, (median [IQR]) | | | |  |  |  |
| SOFA | 5.00 [3.00, 7.00] | 5.50 [3.00, 8.00] | 0.016 | 7.00 [5.00, 9.25] | 9.00 [7.00, 12.00] | <0.001 |
| GCS | 13.00 [8.00, 14.00] | 13.00[8.00, 14.00] | 0.528 | 11.00 [9.00, 14.00] | 9.00 [6.00, 11.00] | <0.001 |
| Mechanical ventilation, (n(%)) | 4468 (77.0) | 685 ( 74.9) | 0.178 | 211 ( 31.0) | 118 ( 37.0) | 0.072 |
| Use of vasopressors, (n(%) | 3511 (60.5) | 525 ( 57.4) | 0.082 | 219 ( 32.2) | 166 ( 52.0) | <0.001 |
| Length of hospital stays, days (median [IQR]) | 2.30 [1.30, 4.50] | 3.90 [1.70, 8.30] | <0.001 | 11.80 [6.60, 21.02] | 8.70 [3.00, 15.70] | <0.001 |

GCS: Glasgow coma scale; SOFA: sequential organ failure assessment;PaCO_2_: partial pressure of carbon dioxide; S_P_O_2_: arterial oxygen saturation; PaO_2_: partial pressure of oxygen.
