# Supplementary material for: Conservative oxygen therapy in critically ill and perioperative period of patients with sepsis-associated encephalopathy
Source: Front Immunol. 2022 Oct 19;13:1035298. doi: 10.3389/fimmu.2022.1035298 (PMC9626799; doi:10.3389/fimmu.2022.1035298)
Supplement: Supplementary file 7 [file Table_4.docx]

| **Supplementary material 4** Multivariate COX analysis of risk factors to hospital mortality in patients with SAE in the MIMIC database | | | | | |
| --- | --- | --- | --- | --- | --- |
|  |  | P | OR | 95.0% CI | |
|  |  |  |  | Lower | Upper |
| Age |  | <0.001 | 1.010 | 1.005 | 1.015 |
| Gender |  | 0.675 | 0.972 | 0.850 | 1.111 |
| Coexisting illness, (n(%)) | | | | | |
| Hypertension |  | 0.020 | 0.777 | 0.629 | 0.961 |
| Diabetes |  | <0.001 | 1.990 | 1.718 | 2.304 |
| Renal |  | 0.002 | 1.247 | 1.082 | 1.438 |
| Site of infection, (n (%)) |  |  |  |  |  |
| Lung |  | 0.324 | 1.139 | 0.879 | 1.476 |
| Catheter |  | 0.147 | 0.709 | 0.445 | 1.129 |
| PaO_2_ (97-339)mmHg |  | 0.625 | 0.928 | 0.689 | 1.251 |
| PaCO_2_ |  | 0.472 | 0.998 | 0.993 | 1.003 |
| Respiratory rate |  | 0.786 | 0.999 | 0.990 | 1.008 |
| S_P_O_2_≥93% |  | 0.017 | 0.679 | 0.494 | 0.934 |
| PaO_2_/FiO_2_ (189-619) |  | <0.001 | 0.377 | 0.319 | 0.447 |
| Creatinine |  | 0.512 | 0.982 | 0.931 | 1.036 |
| Blood urea nitrogen |  | 0.033 | 1.004 | 1.000 | 1.007 |
| Glucose |  | 0.381 | 1.000 | 0.999 | 1.000 |
| SOFA |  | 0.124 | 0.982 | 0.959 | 1.005 |
| PaCO_2_: partial pressure of carbon dioxide; S_P_O_2_: arterial oxygen saturation; PaO_2_: partial pressure of oxygen; SOFA: sequential organ failure assessmen. | | | | | |
