# Supplementary material for: Conservative oxygen therapy in critically ill and perioperative period of patients with sepsis-associated encephalopathy
Source: Front Immunol. 2022 Oct 19;13:1035298. doi: 10.3389/fimmu.2022.1035298 (PMC9626799; doi:10.3389/fimmu.2022.1035298)
Supplement: Supplementary file 8 [file Table_5.docx]

| **Supplementary material 5** Multivariate COX analysis of risk factors to hospital of patients with SAE in the eICU database | | | | | |
| --- | --- | --- | --- | --- | --- |
|  |  | P | OR | 95.0% CI | |
|  |  |  |  | Lower | Upper |
| Age |  | <0.001 | 1.032 | 1.023 | 1.040 |
| Renal disease |  | 0.247 | 0.857 | 0.660 | 1.113 |
| Respiratory rate |  | <0.001 | 1.038 | 1.020 | 1.057 |
| PaO_2_ (97-339) mmHg |  | 0.095 | 0.802 | 0.619 | 1.039 |
| FiO_2_ |  | <0.001 | 5.646 | 3.468 | 9.193 |
| PaO_2_/FiO_2_ (189-619) |  | <0.001 | 0.610 | 0.489 | 0.762 |
| Hemoglobin |  | 0.116 | 0.965 | 0.923 | 1.009 |
| Platelet |  | 0.049 | 1.001 | 1.000 | 1.002 |
| Creatinine |  | 0.589 | 0.977 | 0.900 | 1.062 |
| Blood urea nitrogen |  | 0.232 | 1.003 | 0.998 | 1.008 |
| Glucose |  | 0.899 | 1.000 | 0.997 | 1.002 |
| Lactates |  | <0.001 | 1.066 | 1.039 | 1.094 |
| Use of vasopressors |  | 0.188 | 1.180 | 0.922 | 1.511 |
| SOFA |  | <0.001 | 1.111 | 1.066 | 1.158 |
| PaO_2_: partial pressure of oxygen; FiO_2_: the fraction of inspired oxygen; SOFA: sequential organ failure assessmen. | | | | | |
